# Early-Life Environmental Variation Affects Intestinal Microbiota and Immune Development in New-Born Piglets

**DOI:** 10.1371/journal.pone.0100040

**Published:** 2014-06-18

**Authors:** Dirkjan Schokker, Jing Zhang, Ling-li Zhang, Stéphanie A. Vastenhouw, Hans G. H. J. Heilig, Hauke Smidt, Johanna M. J. Rebel, Mari A. Smits

**Affiliations:** 1 Animal Breeding and Genomics Centre, Wageningen UR Livestock Research, Lelystad, The Netherlands; 2 Laboratory of Microbiology, Wageningen University, Wageningen, The Netherlands; 3 Infection Biology, Central Veterinary Institute, Lelystad, The Netherlands; Max Delbrück Center for Molecular Medicine, Germany

## Abstract

**Background:**

Early-life environmental variation affects gut microbial colonization and immune competence development; however, the timing and additional specifics of these processes are unknown. The impact of early-life environmental variations, as experienced under real life circumstances, on gut microbial colonization and immune development has not been studied extensively so far. We designed a study to investigate environmental variation, experienced early after birth, to gut microbial colonization and intestinal immune development.

**Methodology/Principal Findings:**

To investigate effects of early-life environmental changes, the piglets of 16 piglet litters were divided into 3 groups per litter and experimentally treated on day 4 after birth. During the course of the experiment, the piglets were kept with their mother sow. Group 1 was not treated, group 2 was treated with an antibiotic, and group 3 was treated with an antibiotic and simultaneously exposed to several routine, but stressful management procedures, including docking, clipping and weighing. Thereafter, treatment effects were measured at day 8 after birth in 16 piglets per treatment group by community-scale analysis of gut microbiota and genome-wide intestinal transcriptome profiling. We observed that the applied antibiotic treatment affected the composition and diversity of gut microbiota and reduced the expression of a large number of immune-related processes. The effect of management procedures on top of the use of an antibiotic was limited.

**Conclusions/Significance:**

We provide direct evidence that different early-life conditions, specifically focusing on antibiotic treatment and exposure to stress, affect gut microbial colonization and intestinal immune development. This reinforces the notion that the early phase of life is critical for intestinal immune development, also under regular production circumstances.

## Introduction

Maintenance of general health and prevention of infectious diseases are critically dependent on intestinal homeostasis and proper immune competence. In this regard, early colonization of the gut by microbiota as well as the concomitant development of the intestinal immune system has been proven to be important [1], [2], [3]. Immediately after birth, the intestine is colonized by bacteria derived from maternal and environmental sources [4], [5]. During the early-life period, the composition and diversity of microbiota is unstable and highly influenced by environmental conditions, including the use of antibiotics, exposure to stress, and nutrition, as observed in several recent studies using a variety of experimental conditions and models [6],[7],[8],[9],[10],[11],[12],[13]. A significant difference in the diversity of microbiota has, for example, been identified between naturally-reared piglets and isolator-reared piglets that were separated from each other 24 hours after birth during which period natural colonization occurred [11]. The gut microbiota in the isolator-reared piglets remained very divers and contained a large number of phylotypes, whereas in the naturally-reared piglets the microbial diversity decreased as the piglets developed from neonate (day 5) to adult-like stage (day 56: near maturity) [14]. Naturally rearing is supposed to be superior over growing-up in isolators in terms of health with improved immune development and immune homeostasis [14].

The structural and functional development of the mucosal immune system takes place concomitantly with the early-life microbial colonization of new-borns. There is now significant evidence that the process of immune maturation is influenced by the microbiota that colonize the gut at the early stages of life [15], [16], [17], [18], [19]. A link has been discovered between the functionality of the host immune system and the early-life gut microbiota composition [20], [21], [22]. In addition, it has been observed that components of the early-life environment of piglets, most probably including the gut microbial diversity, affect the number of CD4^+^, CD4^+^CD25^+^ effector T-cells, and CD4^+^CD25^+^Foxp3^+^ regulatory T-cells, as well as the serum IgG antibody response [16]. Furthermore, it has been shown that host species specific microbiota is required for the development of the immune system [1] and that different epithelial cell lineages (e.g. paneth cells) are essential for colonization of commensal microbiota and homeostasis of the intestine [23]. Many of the studies that so far addressed the interaction between microbiota and the host immune system used (extreme) experimental conditions and do not account for the timing and specifics of events during early development encountered by the new born animals under normal production circumstances.

To investigate the impact of early-life variations, as experienced under regular production circumstances, on gut microbial colonization and immune development, we used piglets and environmental conditions common in swine husbandry systems and with similarities to environmental conditions experienced by young infants. In intensive swine husbandry systems, piglets are frequently exposed to antibiotics at young age, mainly to prevent outbreaks of respiratory and intestinal diseases; however, the impact on intestinal health has not yet been described on microbiota and/or gene expression level. On the other hand, new born piglets are also frequently exposed to a number of stressful handlings, including ear-tagging, tail docking, and nail clipping. These treatments are known to cause stress, which is in turn expected to have a negative influence on intestinal health of animals. As shown in rats, acute stress can lead to dysfunction of the intestinal barrier [24].

The objective of this study was to investigate the effects of early-life exposure to antibiotics, and antibiotics in combination with stress at day 4 after birth on gut microbial colonization and immune development under regular production circumstances. The specific objective of this study was to identify and define changes in the composition and diversity of the microbiota in the gut and the concomitant immunological effects in intestinal tissue at day 8 after birth. We studied the effect of an antibiotic exposure on the interaction between host immune development and microbiota, in the presence and absence of stressful management procedures, using community-scale analysis of gut microbiota and genome-wide transcriptome profiling of jejunum and ileum tissue.

## Materials and Methods

### Design

The experiment was conducted with 16 sows (TOPIGS20, GY × NL) and their suckling piglets. The piglets of each sow were divided into 3 treatment groups (T1, T2, and T3) by colour-marks on their back, but stayed during the course of the experiment in the litter with their mother sow (Figure S1). Treatment group 1 (T1) piglets experienced no disturbance and were only handled at the time of drawing blood at day 8. Treatment group 2 (T2) piglets received an injection (subcutaneously in the neck) with 0,1 ml Tulathromycin (dosage was 2.5 mg/kg [1 mL/40 kg] body weight) at day 4 after birth. Treatment group 3 (T3) piglets received an identical injection with 0,1 ml Tulathromycin at day 4 after birth and at the same time the standard management procedures (i.e. docking, clipping and weighing) used at that particular farm (VIC Sterksel, The Netherlands). At day 8 after birth from each litter (n = 16) and from each treatment group (n = 3) 48 piglets were sacrificed by intravenous injection of 0,5–1 ml Euthasol (20% sodium pentobarbital; 200 mg/ml). Intestinal tissues (jejunum and ileum) were taken and immediately frozen in liquid nitrogen. From the same or adjacent locations luminal contents and mucosal scrapings were taken, rinsed (with PBS) and frozen in liquid nitrogen as well.

We performed a power calculation (balanced one-way analysis of variance) in R (version 2.14.0), where the number of treatments was 3, number in each group was 4, significance level was 0.05, and power was 0.8. This resulted in an effect size of 1.07. This means that we can pick-up large effects in gene expression log_2_(Fold Change) ≥1.07 between treatments. For each treatment (T1, T2, and T3) the samples of 4 pools of 4 animals were analysed. This design reduces the effect of maternal genetics. The grouping of piglets in each pool were the same for microbiota, blood and tissue (scrapings) transcriptomic analyses, by pooling 4 animals (Table A in File S1).

### Ethics Statement

This animal experiment was approved by the institutional animal experiment committee “Dier Experimenten Commissie (DEC) Lelystad” (2011077.b), in accordance with the Dutch regulations on animal experiments.

### Microbiota Analysis

Ileal content was not present in all piglets at day 8; therefore, only jejunal content was analysed. Jejunal contents were grouped into 4 pools as described in the ‘Design’ part. Microbial DNA was extracted from 250 mg of the mixture using a faecal DNA extraction protocol adapted from Yu and Morrison [25], as previously described by Salonen *et al*. [26]. After extraction of microbial DNA, the microbial composition was detected by the Pig Intestinal Tract Chip (PITChip) version 2.0. The PITChip is a phylogenetic microarray with 3,299 oligonucleotides based on 16S rRNA gene sequences of 781 porcine intestinal microbial phylotypes, it was designed according to the same principles previously described for the Human Intestinal Tract Chip (HITChip) [27] and the PITChip version 1.0 [28], [29], [30]. The PITCip and the comparable tools targeting the human (HITChip) and mouse (MITChip) intestinal microbiota provide a highly reproducible profile of microbiota composition that has been compared with deep pyrosequencing of 16S rRNA gene fragments [29], [31], [32] and metagenome sequencing of intestinal microbiota [33], indicating comparable phylogenetic resolution and a higher sensitivity of the chip-based analysis. Furthermore, microarray-based analysis is not affected by differences in read-depth per sample as is frequently observed for next generation technology sequencing-based approaches. The protocol for hybridization and analysis of the generated data was performed essentially as described before for the HITChip [27]. Briefly, the bacterial 16S rRNA gene was amplified using the primers *T7prom*-Bact-27-for (5′-TGAATTGTAATACGACTCACTATAGGGGTTTGATCCTGGCTCAG-3′) and Uni-1492-rev (5′-CGGCTACCTTGTTACGAC-3′) [27], [34]. The PCR products were transcribed into RNA and the purified resultant RNA was coupled with CyDye prior to fragmentation and hybridization to the array. Microarray images were processed using Agilent’s Feature Extraction Software version 9.5 (http://www.agilent.com). Data was retrieved from the MySQL (version 5.1) database as describe by Rajilic-Stojanovic [27] and pre-processed using the R (Rx64 2.12.2) microbiome package (http://microbiome.github.com/), settings on default. Although this data is part of a larger dataset, we only provide the data related to day 8 in this study. Diversity of microbial profiles was assessed by calculating the Shannon index of diversity using normalized signal intensities of all probes on the array (http://microbiome.github.com/). Multivariate analysis was applied for PITChip data interpretation. In order to relate changes in total microbial composition to environmental variables, redundancy analysis (RDA) was used as implemented in the CANOCO 4.5 software package (Biometris, Wageningen, The Netherlands) [35]. Treatment classes were introduced as environmental (explanatory) variables. The signal intensities for 151 genus-level phylogenetic groups targeted by the PITChip were used as response variables. RDA was performed focusing on inter-sample correlation, and the Monte Carlo Permutation test was applied [36] to decide whether treatment had statistically significant influence on the microbial composition. The unrestricted permutation option (since the experiment had a randomized design) that yields completely random permutations was employed [37]. Treatment was considered to significantly affect microbial composition with p-values <0.05. Triplot diagrams were generated using CanoDraw for Windows. To test the variation of an individual microbial group between treatments we performed a Mann-Whitney-Wilcoxon signed rank test in R (version 2.14.0) with multiple testing corrections (Benjamini Hochberg).

### Microarray Analysis

#### RNA extraction blood

Blood was sampled using the PAXgene Blood RNA tube from PreAnalytiX (a Qiagen/BD company). Tubes were thawed for 2 hours at room temperature (RT). Subsequently tubes were centrifuged at 3,200× g for 10 minutes at RT and the supernatant was discarded. The pellet was suspended in RNase-free water and tubes were centrifuged as described above. The pellet was washed again with RNAse-free water by repeating the last two steps. After discarding the supernatant the pellet was dissolved in 1 ml TRIzol reagent (Invitrogen). After 5 min at room temperature 0.2 ml of chloroform was added, the tubes were mixed vigorously for 15 seconds. After 2 min at RT, tubes were centrifuged for 15 min at 7,000× g. The watery phase containing the RNA was isolated and mixed with 0.5 ml of isopropanol. After centrifugation at 12,000× g for 10 minutes at RT, RNA pellets were washed with 75% (v/v) ethanol and dissolved in RNase-free water. Quality control was performed with the Bioanalyzer from Agilent.

#### RNA extraction tissue

Total RNA was extracted from 50 to 100 mg tissue samples of jejunum and ileum. The jejunum and ileum samples were homogenized using the TisuPrep Homogenizer Omni TP TH220P) in TRizol reagent (Life Technologies) as recommended by the manufacturer with minor modifications. The homogenized tissue samples were dissolved in 5 ml of TRizol reagent. After centrifugation the supernatant was transferred to a fresh tube. Subsequently a phase separation with chloroform was performed as described by the manufacturer. The RNA was precipitated and dissolved, and quantified by absorbance measurements at 260 nm.

#### RNA labelling, hybridization, scanning and feature extraction

Labelling was done as recommended by Agilent Technologies using the One-Color Microarray-Based Gene Expression Analysis Low input Quick Amp Labeling. The input was 200 ng of total RNA and 600 ng of labelled cRNA was used on the 8 pack array.

Hybridization was performed as described in the One-Color Microarray-Based Gene Expression Analysis Low input Quick Amp Labeling protocol from Agilent in the hybridization oven (G2545A hybridization Oven Agilent Technologies). The hybridization temperature was 65°C with rotation speed 10 rpm for 17 hours. After 17 hours the arrays were washed as described in the One-Color Microarray-Based Gene Expression Analysis Low input Quick Amp Labelling protocol from Agilent.

The porcine Agilent microarray slides, G2519F *Sus scrofa* (035953; V2∶026440), harbouring 43,803 probes, were used and scanned using the DNA microarray scanner with Surescan high resolution Technology (Agilent Technologies). Agilent Scan Control with resolution of 5 µ, 16 bits and PMT of 100%. Feature extraction was performed using protocol 10.7.3.1 (v10.7) for 1 colour gene expression.

#### Data loading and statistical analysis

The files generated by the feature extraction software were loaded in GeneSpring GX 12 (also available in GEO, accession number; GSE53170, platform; GPL18045), in which a log2-transformation and quantile normalization were performed. After quantile normalization, quality control was performed and 2 samples were taken out, one sample of jejunum and one of ileum. The remaining 22 samples were analysed by principle component analysis, and a similar approach was followed for the 12 blood samples.

Subsequently, the data was filtered based on the level of expression in which only the (20–100)^th^ percentile was included and control probes if present were removed, resulting in 28,953 probes for the tissue samples, and 20,832 probes for the blood samples. Thereafter (multiple) probes were mapped to genes if possible, resulting in 21,660 probes/genes for tissue samples, and 15,296 probes for the blood samples. To calculate whether the difference between treatments was significant a 2-way ANOVA with multiple testing correction (Benjamini-Hochberg) was performed within GeneSpring, where we compared the following groups for blood, jejunum and ileum: T3 vs. T1, T2 vs. T1, and T3 vs. T2.

#### Functional annotation clustering

Functional annotation clustering analyses were performed with Database for Annotation, Visualization and Integrated Discovery (DAVID, version 6.7 [38], [39]). For each comparison, T3 vs. T1, T2 vs. T1, and T3 vs. T2, analyses were performed with lists of significantly up- and down-regulated genes (after filtering and mapping of probes). An overview of the number of up- and down-regulated genes is available in Table B in File S1.

#### Gene set enrichment analysis (GSEA)

GSEA [40], [41] was performed separately for ileum and jejunum. The following settings were different from the default settings: permutations were performed on the gene set, chip platform was set to gene symbol. Six gene set databases (v3.0) were loaded for analysis, including the three Gene Ontology related gene sets biological processes, molecular function and cellular component, and three pathway related gene sets, BioCarta, Reactome and KEGG.

#### InnateDB interactions

Interactions were extracted from innateDB [42] and loaded into CytoScape (v2.8.1) [43], [44]. To represent the cell location for all genes in the network, the Cerebral plugin was used [45].

#### Quantative RT-PCR

For the quantification of expression levels based on array data, cDNA was made using random hexamer primers and reverse transcriptase, and qRT-PCR was performed targeting genes encoding IL-1βb [46], IL-6 [46], IL-8 [46] and TIMP1 [47] with on-line detection using Syber Green PCR Master Mix (Applied Biosystems, Foster City, CA, USA) in an ABI 7500 Real-Time PCR system (PE Applied Biosystems, Foster City, CA, USA). Quantitative results were determined and normalized with GAPDH gene expression (Figure S2). For the quantification, a standard curve of a plasmid containing the cytokine gene of interest or GAPDH in pGEM-T easy (Promega Benelux b.v. Leiden, The Netherlands) was used.

## Results

### Microbiota analyses

**Figure 1:**
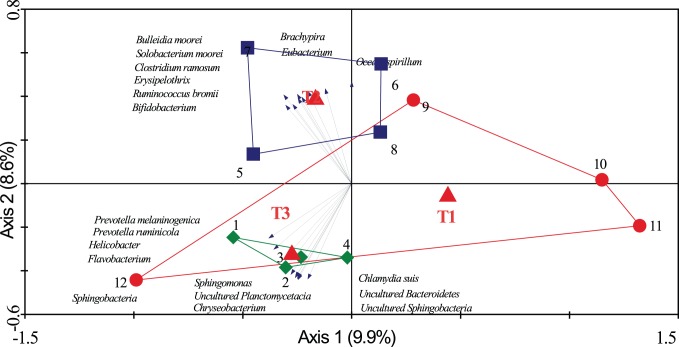
Triplot for RDA analysis of jejunal microbiota composition. Nominal environmental variables T1, T2 and T3 are represented by red triangles (▴). Samples are grouped by treatment: T1 (red; ○), T2 (blue; □) and T3 (green; ◊), each symbol represents a pool of four pigs, and numbers represent pool identity number. Microbial groups contributing at least 60% to the explanatory axes are represented as vectors. Both axes together explain 18.5% of the total variance in the dataset.

**Figure 2:**
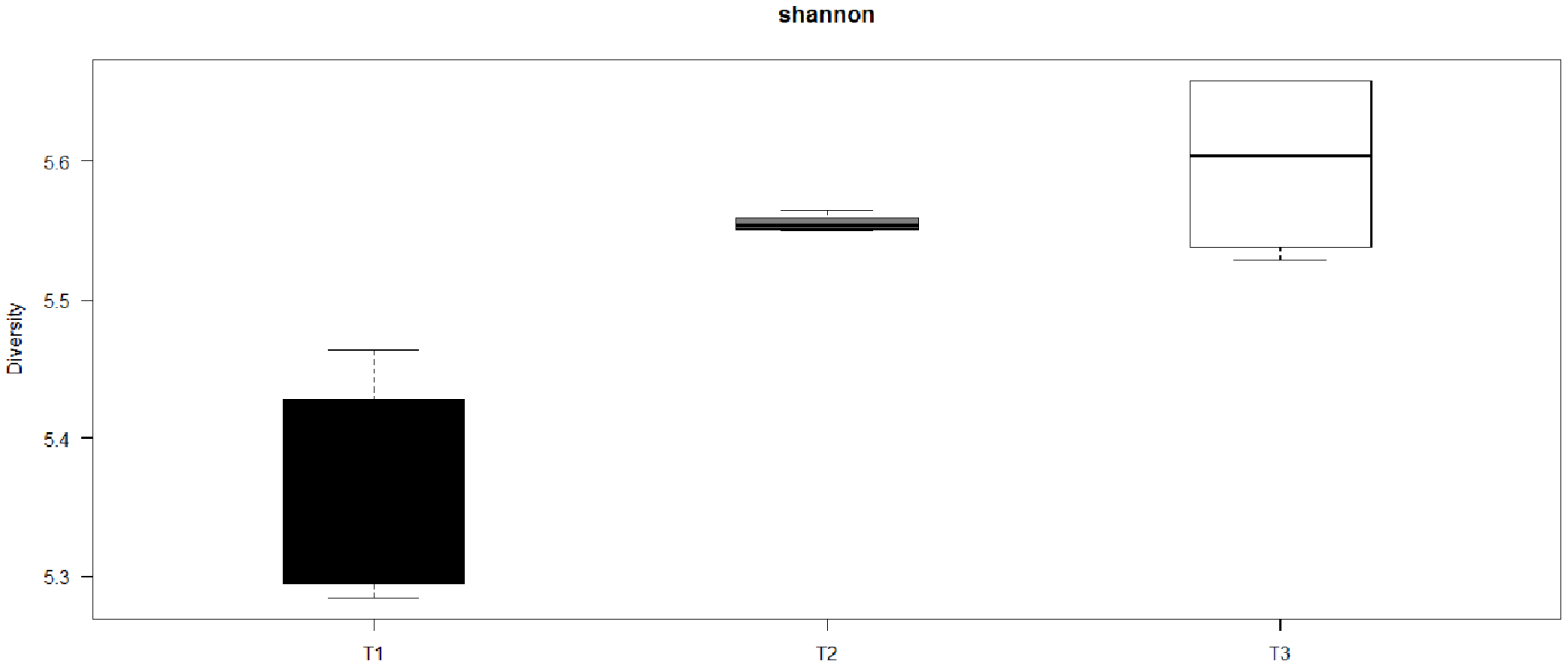
Diversity in microbiota in the three treatment groups. The Shannon index (y-axis) was calculated for all three treatments (T1, T2, and T3) (x-axis).

The PITChip was used to evaluate the impact of antibiotic treatment with or without routinely stressful management on jejunal microbiota. The most dominant phylum in all samples was the Firmicutes, followed by Proteobacteria, Bacteriodetes, Spirochaetes, and Actinobacteria (Table C in File S1), whereas, the relative abundances of other phyla were not above 1%. Multivariate redundancy analysis of PITChip2 profiles at the approximate genus-level showed that samples of T3 separated from most samples of T1 along the first canonical axis except for sample 12, and samples of T2 separated from those of T1 along the second canonical axis (Fig. 1). Both axes together explained 18.5% of the variance in microbiota composition. Subsequently, univariate analysis was used to identify microbial groups that significantly changed between treatments (Table 1). In the comparison of T2 versus T1, the relative contribution of *Bifidobacterium-*like, *Erysipelotrichi-*like, *Eubacterium-*like, *Faecalibacterium prausnitzii-*like, and *Solobacterium moorei*-like bacteria increased, whereas *Bacillus-*like and *Staphylococcus aureus-*like bacteria strongly decreased. In contrast, uncultured *Prevotella* group (i.e. a genus-level phylogenetic group comprising exclusively environmental sequences and no cultured representatives) increased in its relative abundance in T3 compared to T1 piglets. When comparing T3 versus T2, multiple microbial groups in different phyla were significantly different, including groups belonging to the Actinobacteria, Fibrobacteres, Firmicutes, Proteobacteria and Spirochaetes (see Table 1 for more detailed information). Microbial diversity (Shannon index based on probe-level profiles) was significantly higher in T2 and T3 than in T1 (*p*<0.01); however, no significant difference was observed between T3 and T2. (*p*>0.05) (Fig. 2).

**Table 1:** Genus-level phylogenetic groups changed in T2 and/or T3.

Microbial groups	T2 vs. T1		T3 vs. T1		T3 vs. T2		ARC^2^		
	*P* value	C.P^1^	*P* value	C.P	*P* value	C.P	T1	T2	T3
Actinobacteria									
* Actinobacteria*									
* Bifidobacterium et rel.*	**0.03↑** 3	0.48	0.34	0.51	**0.03↓**	0.1	0.27±0.08	0.48±0.08	0.34±0.04
* Collinsella*	0.49	0.71	0.11	0.51	**0.03↓**	0.1	0.19±0.04	0.24±0.09	0.14±0.01
* Olsenella et rel.*	0.34	0.67	0.2	0.51	**0.03↓**	0.1	0.18±0.04	0.24±0.09	0.14±0.02
Bacteroidetes									
* Bacteroidetes*									
Uncultured *Prevotella*	0.2	0.64	**0.03↑**	0.33	0.89	0.96	0.2±0.03	0.29±0.08	0.32±0.05
Fibrobacteres									
* Fibrobacteres*									
* Fibrobacter succinogenes et rel.*	0.34	0.67	0.34	0.51	**0.03↓**	0.1	0.58±0.23	0.7±0.11	0.43±0.02
Firmicutes									
* Bacilli*									
* Allofustis et rel.*	0.49	0.71	0.34	0.51	**0.03↑**	0.1	0.26±0.04	0.25±0.01	0.28±0
* Bacillus et rel.*	**0.03↓**	0.48	0.06	0.51	0.34	0.52	0.39±0.01	0.34±0.02	0.36±0.02
* Carnobacterium et rel.*	1	1	0.34	0.51	**0.03↑**	0.1	0.64±0.16	0.57±0.06	0.68±0.06
* Staphylococcus aureus et rel.*	**0.03↓**	0.48	0.69	0.85	**0.03↑**	0.1	0.22±0.03	0.19±0.01	0.22±0.01
* Streptococcus suis et rel.*	0.06	0.54	0.89	1	**0.03↓**	0.1	0.55±0.16	0.83±0.09	0.56±0.1
* Clostridium cluster IV*									
* Anaerotruncus et rel.*	0.89	0.94	0.34	0.51	**0.03↑**	0.1	1.27±0.35	1.16±0.14	1.44±0.07
* Faecalibacterium prausnitzii et rel.*	**0.03↑**	0.48	0.2	0.51	0.2	0.38	0.22±0.09	0.37±0.04	0.31±0.08
* Ruminococcus bromii et rel.*	0.06	0.54	0.34	0.51	**0.03↓**	0.1	0.27±0.06	0.41±0.06	0.31±0.02
* Clostridium cluster XI*									
* Anaerovorax et rel.*	0.49	0.71	0.34	0.51	**0.03↑**	0.1	3.35±0.59	3.07±0.18	3.57±0.09
* Clostridium cluster XIII*									
* Peptoniphilus et rel.*	0.34	0.67	0.89	1	**0.03↑**	0.1	1.02±0.19	0.9±0.09	1.1±0.08
* Clostridium cluster XIVa*									
* Clostridium sphenoides et rel.*	0.49	0.71	0.34	0.51	**0.03↑**	0.1	0.69±0.15	0.62±0.07	0.8±0.04
* Ruminococcus obeum et rel.*	0.69	0.82	0.2	0.51	**0.03↑**	0.1	2.45±0.76	2.43±0.47	3.4±0.39
* Clostridium cluster XV*									
* Eubacterium et rel.*	**0.03↑**	0.48	0.89	1	**0.03↓**	0.1	0.15±0.05	0.26±0.03	0.16±0.03
* Clostridium cluster XVII*									
* Catenibacterium et rel.*	0.11	0.54	0.49	0.7	**0.03↓**	0.1	0.22±0.11	0.4±0.16	0.23±0.01
* Erysipelotrichi*									
* Solobacterium moorei et rel.*	**0.03↑**	0.48	0.49	0.7	**0.03↓**	0.1	0.11±0.05	0.22±0.04	0.14±0.02
Proteobacteria									
* Betaproteobacteria*									
* Bordetella et rel.*	0.11	0.54	0.89	1	**0.03↓**	0.1	0.43±0.17	0.73±0.18	0.41±0.05
* Oxalobacter et rel.*	0.11	0.54	1	1	**0.03↓**	0.1	0.12±0.04	0.21±0.07	0.11±0.01
* Sutterella wadsorthia et rel.*	0.11	0.54	0.89	1	**0.03↓**	0.1	0.12±0.06	0.24±0.09	0.11±0.01
* Gammaproteobacteria*									
* Psychrobacter et rel.*	0.2	0.64	1	1	**0.03↓**	0.1	0.31±0.08	0.41±0.07	0.33±0.01
* Ruminobacter amylophilus et rel.*	0.11	0.54	1	1	**0.03↓**	0.1	0.24±0.08	0.34±0.06	0.23±0.01
Spirochaetes									
* Spirochaetes*									
* Treponema et rel.*	0.49	0.71	0.89	1	**0.03↓**	0.1	1.31±0.32	1.46±0.13	1.19±0.09
* Turneriella*	0.2	0.64	1	1	**0.03↓**	0.1	0.2±0.13	0.32±0.07	0.15±0.01

1 C.P: P value corrected for multiple testing according to the procedure of Benjamini-Hochberg.

2 ARC: average relative contribution [%] of a microbial group. Values represented means ± SD. The microbial groups with a relative abundance lower than 0.1% in all three treatments are not shown.

3 “**↑**” or “**↓**” indicates whether the average relative contribution of the microbial group was increased or decreased.

### Transcriptomic Analysis

**Figure 3:**
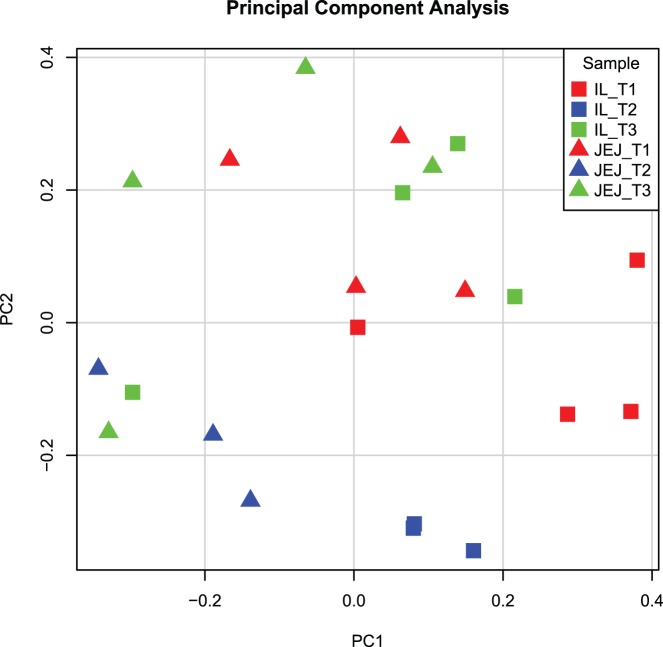
Principal Component Analysis on microarray data of intestinal tissue transcriptomes. A principal component analysis was performed on the 22 tissue transcriptome datasets which remained after quality control. All treatments (T1: red, T2:blue, and T3:green) are displayed for both jejunum (triangles) and ileum (squares). The x-axis depicts principal component 1 and the y-axis depicts principal component 2. Abbreviations: IL; Ileum, JEJ; Jejunum.

**Figure 4:**
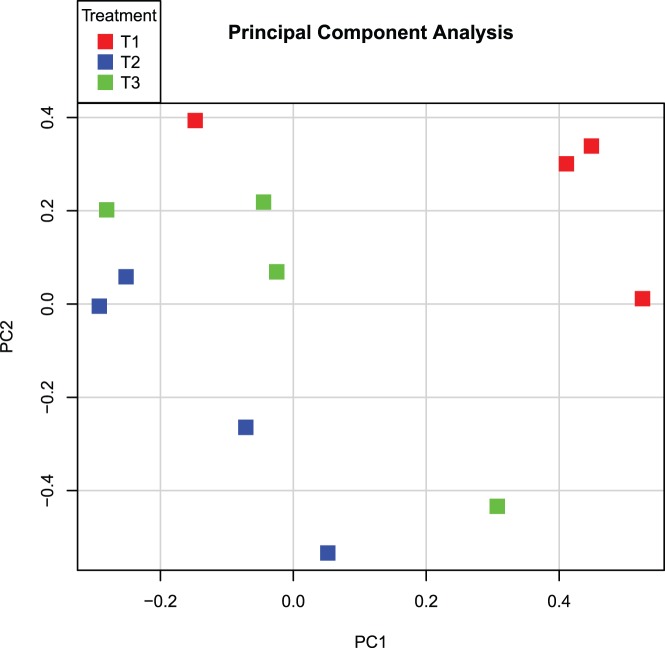
Principal component analysis of blood transcriptomes. A principal component analysis was performed on the 12 blood transcriptome data sets which remained after quality control. Treatment 1 (T1) is depicted in red, T2 in blue, and T3 in green. The x-axis depicts principal component 1, whereas the y-axis depicts principal component 2.

To investigate the impact of the different treatments on the host, genome-wide gene expression was measured in intestinal tissue and blood samples. Initially, Principal Component Analysis (PCA) was performed to get more insight into the variability in blood and tissue transcriptome data and to get a visual inspection of the quality of the data. Representing transcriptome data by PCA showed clear aspects of quality control and outlier arrays and hybridization samples can be spotted and taken out based on their gene expression profile and hybridization pattern to ‘control’ probes. Because both intestinal tissues (jejunum, ileum) origin from the same ancestor cells, similar responses to the treatments were expected, and therefore jejunum and ileum data were analysed simultaneously (Fig. 3), whereas the data of blood samples were loaded separately (Fig. 4). For tissue-derived data, clustering occurred of similar treatments (red; T1, blue; T2, and green; T3) as well as similar tissues (squares; ileum, and triangles; jejunum). However treatment 3 samples were more dispersed compared to treatment 1 and 2, albeit grouping more closely with T1 samples (Fig. 3). Also, the different blood samples clustered, however, these clusters were dispersed when looking at principal component 1 (PC1; Fig. 4). This large variation within the treatment groups hinted that there were no significant differences in gene expression in blood cells between the treatments.

To investigate the effect of the three treatments in jejunum, ileum, and blood, an ANOVA was performed. All probes/genes which were characterized by p_cor_ <0.05 and Fold Change>|1.5| in at least one of the six comparisons were taken for further functional and enrichment analysis (Table B in File S1). No differences were observed in blood, whereas in jejunum and ileum multiple probe signals (up to 80 genes) significantly differed between the treatment groups. In general more probe signals differed significantly in ileum compared to jejunum.

The significantly up- and down-regulated genes were used as input for functional analysis. First, DAVID functional annotation clustering was performed resulting in multiple groups with a significant Enrichment Score (ES). The results are presented in Tables 2–4 (ES>1), where Table 2 shows the differences between T2 and T1, Table 3 between T3 and T1, and Table 4 between T3 and T2. In addition, Gene Set Enrichment Analysis (GSEA) was performed without pre-filtering of genes on e.g. p-value or fold change, so that all probes/genes were used as input. The GSEA analysis resulted in the identification of similar biological processes affected by the treatments as observed by the DAVID analysis (Table D and E in File S1). The main findings were that immune related processes appeared to be dominantly influenced by the treatments, for example cytokine/chemokine related processes differed between the treatments in both tissues. This was also verified for three genes (IL1B, IL6 and TIMP2) with qPCR, where we observed a trend between the qPCR and transcriptomics datasets (Figure S2).

**Table 2:** Table **2.** Functional analysis of genes differentially expressed between treatment 2 versus 1.

	DOWN	
JEJUNUM	#	Name
	1	chemotaxis
	2	cytokine activity
	3	chemokine activity
	4	regulation of secretion/immune effector process
	5	cell migration/motion (leukocyte)
**ILEUM**	**#**	**Name**
	1	cytokine activity
	2	chemotaxis
	3	second-messenger-mediated signaling (cAMP)
	4	chemokine activity
	5	response to bacterium/regulation of systemic process

**Table 3:** Functional analysis of genes differentially expressed between treatment 3 versus 1.

	DOWN		UP	
JEJUNUM	#	Name	#	Name
	1	chemotaxis	**1**	nucleotide binding
	2	cytokine activity	**2**	membrane fraction
	3	extracellular region	**3**	ATP binding
	4	chemokine activity	**4**	
	5	second-messenger-mediated signaling (cAMP)	**5**	
**ILEUM**	**#**	**Name**	**#**	**Name**
	1	response to wounding/defense response	**1**	nucleotide binding
	2	cytokine activity	**2**	positive regulation of catalytic activity/signaling cascade
	3	chemotaxis	**3**	plasma membrane
	4	extracellular region	**4**	
	5	chemokine activity	**5**	

**Table 4:** Functional analysis of genes differentially expressed between treatment 3 versus 2.

	DOWN	
JEJUNUM	#	Name
	**1**	chemotaxis
	**2**	nucleotide binding
	**3**	
	**4**	
	**5**	
**ILEUM**	**#**	**Name**
	**1**	Immunoglobulin
	**2**	nucleotide binding
	**3**	plasma membrane
	**4**	nucleotide/ATP binding
	**5**	positive regulation of catalytic activity/signaling cascade

**Figure 5:**
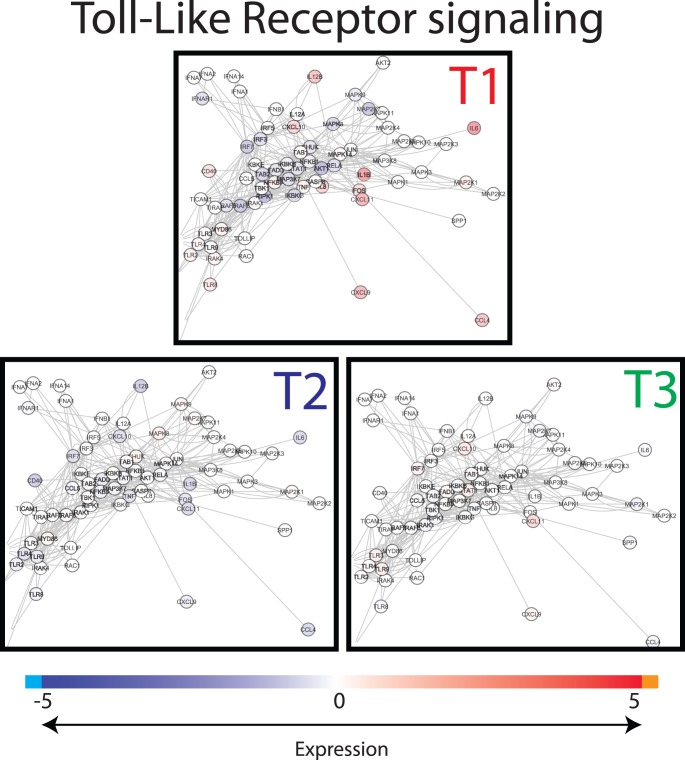
Snapshot of Toll-like receptor network. Interactions of genes involved in the Toll-like receptor (TLR) pathways were extracted from innateDB and visualized in CytoScape. Nodes (genes) are coloured by their expression, where blue is low expression and red is high expression (see legend on top). Abbreviations used: T1; Treatment 1, T2; Treatment 2, T3; Treatment 3.

**Figure 6:**
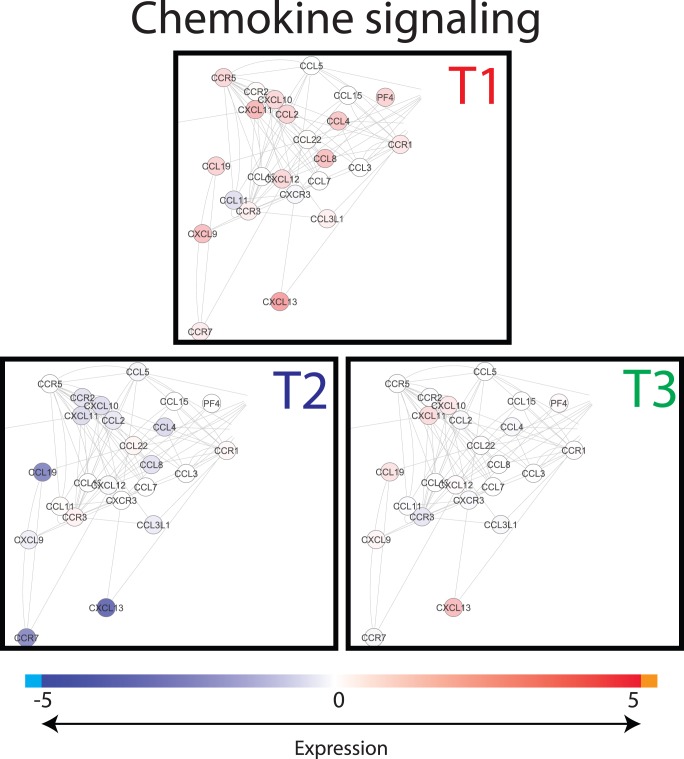
Snapshot of chemokine network. Interactions of genes associated with chemokine pathways were extracted from innateDB visualized in CytoScape. Nodes (genes) are coloured by their expression, where blue is low expression and red is high expression (see legend on top). Abbreviations used: T1; Treatment 1, T2; Treatment 2, T3; Treatment 3.

**Figure 7:**
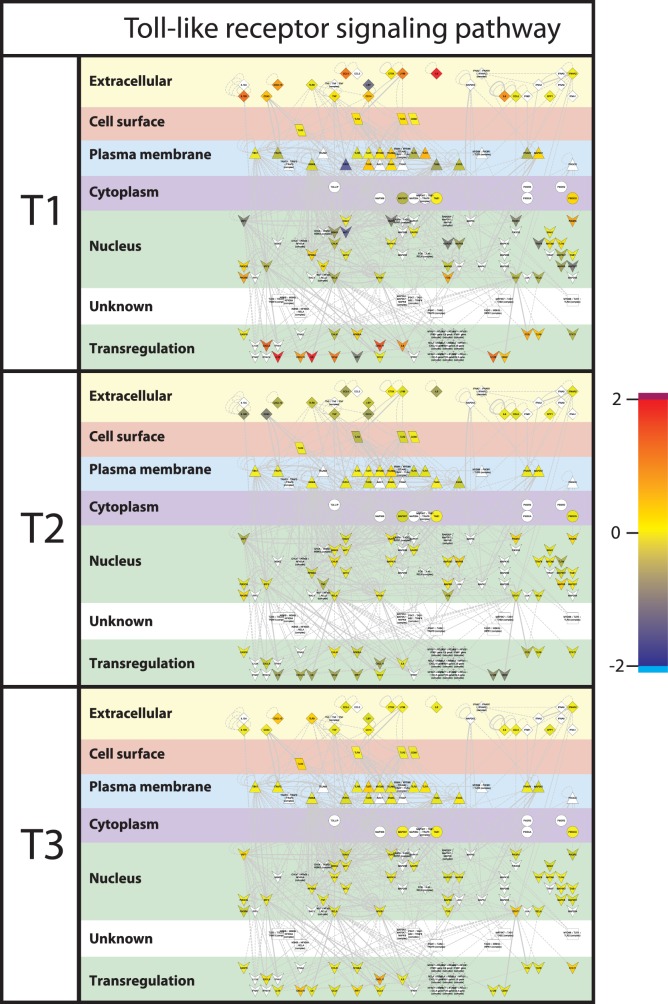
Toll-like receptor signalling pathway. Interactions of genes associated with Toll-like receptor pathways were extracted from innateDB [42] and visualized in CytoScape. Nodes (genes) are coloured by their expression, where blue is low expression and red is high expression. Furthermore, localization of gene products within the cell was predicted using Cerebral [45]. Abbreviations used: T1; Treatment 1, T2; Treatment 2, T3; Treatment 3.

**Figure 8:**
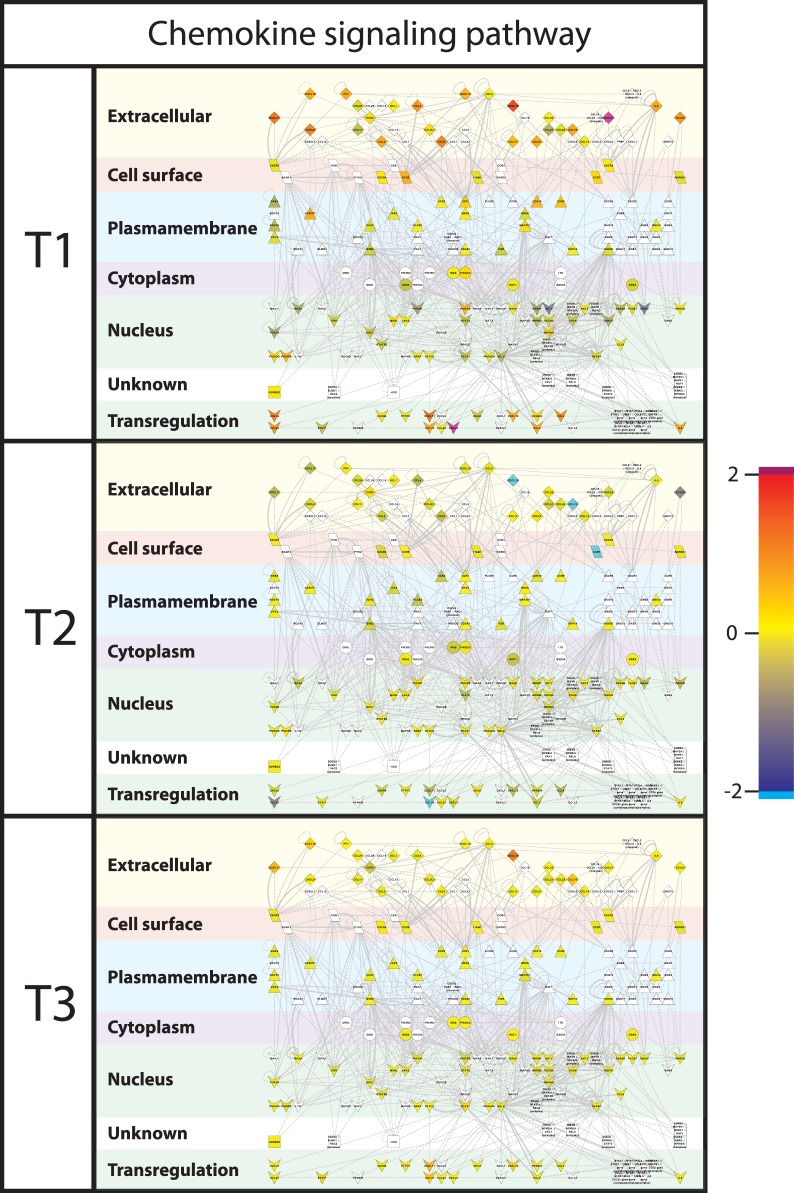
Chemokine signalling network. Interactions of genes associated with chemokine pathways were extracted from innateDB [42] and visualized in CytoScape. Nodes (genes) are coloured by their expression, where blue is low expression and red is high expression. Furthermore, localization of gene products within the cell was predicted using Cerebral [45]. Abbreviations used: T1; Treatment 1, T2; Treatment 2, T3; Treatment 3.

To visualize the differences in gene expression between the treatments, gene interactions networks associated with the dominant processes identified from the functional analysis by DAVID and GSEA were extracted from InnateDB, and the gene expression values were superimposed on these networks. Major differences were found in gene expression levels between the treatment groups for the chemokine signalling network (Fig. 5) and for the Toll-like Receptor (TLR) signalling network (Fig. 6). When focusing on the cellular location of the differentially expressed gene-encoded products of the cytokine and TLR networks, it is remarkable to see that especially the products situated extracellularly, on the cell surface, and the plasma membrane (Fig. 7 and 8), differed between the treatment groups. In T2 and T3 piglets lower expression was observed in these 2 networks compared to the untreated (T1) piglets. Further differentiation could be made between T2 and T3, where in T2 expression is lower compared to T3. In other words, expression of immune associated genes was high in T1, low in T2, and intermediate in T3.

## Discussion

Effects of the different treatments on day 4 after birth were clearly visible at day 8 after birth, for both community-scale microbiota data and genome-wide transcriptomic data. The current study shows effects of early-life environmental variation on microbial colonization and immune development in the gut of piglets that were kept under regular-production circumstances. Here, we will focus specifically on comparison of T1 versus T2/T3 animals, for both microbiota and gene expression.

### Comparison of Microbiota Composition and Diversity between Treatment Groups

The diversity of microbiota as measured by the PITChip in jejunum digesta of T1 animals was significantly lower compared to that in the T2 and T3 animals. This study shows that a single antibiotic dose, administered at day 4 after birth, is able to modulate the microbial community for an extensive time. The antibiotic used in this study is regularly applied at pig farms with a one-time administration because of the long half-life (70 h) of the product and elimination half-life was approximately 6–8 days (details on website European Medicines Agency). This is in line with previous observations, which showed that microbiota composition and diversity was affected for at least five weeks in new-born piglets after a single dose of parenteral amoxicillin treatment [48]. Different methodologies were used in our study as compared to Janczyk *et al.* to assess microbial diversity, i.e. microarray- and denaturing gradient gel electrophoresis (DGGE)-based profiling of PCR-amplified 16S rRNA gene fragments. Nevertheless, we observed that the trends in diversity are comparable, also with those obtained by next generation technology sequencing, albeit at different absolute values [29], [30], [31], [32], [33]. Also the bacterial composition is not likely to return to its initial state, because the gastrointestinal tract undergoes a rapid dynamic development.

In this experiment the antibiotic treatment (T2) caused a detectable change in relative abundance of most early gut colonizers. Among these colonizers, the abundance of all anaerobic bacteria including *Bifidobacterium, Eubacterium, F. prausnitzii,* and *S. moorei* increased, whereas facultative bacteria such as *S. aureus* decreased in quantitative terms. These results may contradict some previous reports, it has been reported that a range of different antibiotics (including Amoxicillin, Ciprofloxacin, Augmentin and Trimethoprim) can reduce *Bifidobacterium* in adult faecal microbiota [49], [50], however, a strong increase of *Bifidobacterium* relative abundance was observed in this study in response to the antibiotic tulathromycin. One possible reason could be that certain antibiotic resistant *Bifidobacterium* strains, such as *B. longum* and *B. catenulatum* [51], can survive under the tulathromycin antibiotic selection. Another explanation could be a difference in the activity of antibiotics in the intestinal lumen compared to the colon. Nevertheless, a higher microbial diversity in T2 detected in this study may indicate a relatively complex microbiota of T2 animals compared to T1 animals. In the complex gut microbial system, facultative bacteria, such as *S. aureus* can not withstand the competition and environmental changes brought by the anaerobes, as shown in young infants [52]. In addition, *S. aureus* colonization has been reported negatively correlated to certain antibiotic treatments at 6 months of age in infants [53], [54], [55]. Thus, the reduction of *S. aureus* in T2 could be attributed to the acceleration of complex anaerobic microbiota establishment by the antibiotic and/or its sensibility to the antibiotic. On the contrary, we did not found a notable change of *S. aureus* between T3 and T1 animals. It appeared that the antibiotic effect was counteracted by stress management, because the microbial groups observed in T2 animals were not observed in T3 animals. Interestingly, this was also observed for *Bifidobacterium, Eubacterium,* and *S. moorei*.

In addition to the microbial groups discussed above, there are 20 microbial groups that only showed difference between T2 and T3 in this study. This result may be attributed to a yet unknown combined effect of antibiotic and management treatment (causing stress), which may involve complex host-microbial interactions in the gut.

The results from these microbiota analyses show that microbial diversity and composition are affected by the different treatments. Earlier findings of Schmidt *et al.* also showed that continuous microbial exposure during early life stages is required for the development of a stable gut microbiota. However, it should be noted that, contrary to our study, Schmidt *et al.* measured the mucosa-adherent microbiota [11]. In the present study, piglets were housed under field conditions of commercial intensive farming systems that correspond to ‘natural’ and continuous exposure to microbial species. Compared to the control T1 group, the administration of tulathromycin caused a clear increase in microbial diversity, leading to a situation which is described by others as “more chaotic” [11]. The microbiota of ‘natural’ colonized piglets is characterized by a high abundance of lactobacilli and low abundance of microbial species related to pathogens. Such a ‘natural’ microbiota composition and diversity has been proposed to be important for immune homeostasis [14].

At the phylum level similar relative abundance levels of microbial groups were observed between the different treatment groups (Table C in File S1), which is in contrast to the observation of Mulder *et al.* (2009) where antibiotic treatment resulted in a severe reduction in abundance of Firmicutes and increase of Bacteriodetes and Proteobacteria compared to the outdoor situation [14]. However, in the study of Mulder a cocktail of antibiotics was administered daily from day 1 to 28 of age, as opposed to a single dose of one antibiotic at day 4 in this study. Furthermore the phylum level data in Mulder *et al*. represents an overall abundance of (mucosa-adherent) microbial groups in the whole experiment, thus including different time-points, which makes it more difficult to compare with our data. Despite of the differences in experimental setup, it is known that both mucosal and luminal microbiota are able to interact with the host [56], however, luminal microbiota might do this more indirectly. Both studies show that the microbial diversity is affected by the antibiotic treatment, and concomitantly gene expression data showed shifts in communicative/immune processes at day 5 and/or our day 8 data.

### Comparison of Gene Expression in Host Blood and Intestinal Tissues

Simultaneous to the microbiota sampling, gene expression measurements were performed of intestinal tissue (scrapings) at the same location (in case of jejunum). In addition, blood transcriptome analysis was performed in order to investigate whether cross-talk between host and microbiota during this neonatal period induced systemic changes. However, the lack of significant differences between the blood samples of the treatment groups suggests that affected immunological processes are still local 4 days after treatment. When comparing the two different tissues analysed here, more genes were differentially expressed, either up or down, in ileum in contrast to jejunum. The dominant processes that were affected by the treatments (T2 or T3) are mostly involved in immunological processes and the genes involved were down-regulated in comparison to T1 animals. In connection with this, we observed that most of the biological processes affected by the treatments (T2 or T3) are involved in various immune functions. The transcriptomic data suggest that the immunological development after birth can be influenced by external factors (e.g. antibiotics and/or stress), possibly by modulating the microbial colonization of the gut [57]. Surprisingly, the top 5 processes in the functional analyses were all of communicative nature for both jejunum and ileum, including “Chemotaxis” (cell or bacterial movement towards a chemical or protein); “chemokine activity”; and “cytokine activity”, the latter of which are associated with immune-modulating activities and involved in (intra)cellular communication. The term “cyclic adenosine monophosphate (cAMP) signaling” is also associated with cell communication (intracellular signal transduction, including suppression of regulatory T cells [58]), whereas “extracellular region” is associated with the space external to the outermost structure of a cell (e.g. plasma membrane). To investigate the above described immunological differences in more detail, a systems approach was applied, by extracting gene-gene interaction networks (InnateDB) for two immunological processes, namely chemokine and Toll-Like Receptor (TLR) signalling. These two networks were chosen because expression of many of the genes that were higher in T1 compared to T2 or T3 piglets belonged to these networks. Furthermore, chemokines play a major role into guiding the migration of cells, and TLRs play a key role in innate immunity and recognition of conserved microbial structures [59]. The data presented here suggests that in the intestine of T1 piglets more intensive immunological communication occurs compared to T2 and T3 piglets, which is reflected by high expression of chemokine and TLR receptors as well as their respective ligands. We hypothesize that the global down-regulation in the antibiotic treated animal (T2/T3) compared to the control (T1) piglets could be that due to the high diversity and more “chaotic” microbial population in the gut of piglets treated with the tulathromycin antibiotic. Similar observations (down regulation vs chaotic/high diversity) were observed by the Kelly group [8], [11], [14].

### Interactions between Host and Microbiota

The genes associated with the immunological processes that differ between the treatment groups are more strongly expressed in T1 animals compared to T2 and T3 animals, which might be due to the observed lower diversity in microbiota and the abundance of specific (non-pathogenic) bacterial species (e.g. lactobacilli). We observed a trend towards higher abundance of lactobacilli in T1 piglets compared to the treatment groups (T1; 18%, T2; 14%, and T3; 12%). A higher abundance of lactobacilli could lead to more cross-talk between these abundant microbiota and host (immune) cells, the more so because it has previously been shown that different lactobacilli strains can modulate the expression of immune pathways [60], [61]. It is known that communication between and within intestinal cells as well as between microbiota and intestinal cells is crucial to set up a proper development (shaping) and functioning of the immune system [62]. In case of early antibiotic treatment and a concomitant increase of microbial groups related to (intestinal) pathogens, it could be hypothesized that due to the antibiotic treatment (too much) immunological tolerance is build-up during early life stages against species related to pathogens, resulting in an inefficient immune response later in life upon pathogenic challenge. This hypothesis regarding the presumed interaction between microbiota and immune development is in line with previous conclusions [7], [10], [21], [22], [63]. We intend to study this specifically in a further project.

### General Conclusion

Different early-life exposure under real-life circumstances, no antibiotic treatment, antibiotic treatment, or antibiotic and stressful treatment, affect the microbial colonization and immune development of piglets. The results shown here suggest a different immune development due to the early life treatments in these piglets. The next step will be to investigate which effect the early-life environment variation has on the immune competence later in life.

## Supporting Information

Figure S1 **Depicted is the experimental design, for each sow (n = 16) piglets are divided over the three treatments (T1, T2, and T3).** At the day of sampling 16 piglets, 1 of each sow are taken for analysis, for each treatment.
(EPS)

Figure S2 **In the x-axis the qPCR signal is depicted and in the y-axis the normalized expression value is depicted.** Three genes are depicted IL1B (upper panel), IL6 (middle panel), and TIMP1 (lower panel).
(EPS)

File S1 **Supporting Tables.** Table A, Pools for microbiota and transcriptomic analyses. Table B, Number of up- or down-regulated probes and genes at day 8 after birth when comparing the different treatments (T1, T2, and T3). Numbers of annotated genes are given in parentheses. Table C, Phylum level bacteria changed in T2 and T3. Table D, Summary of most prominent terms of GSEA analysis in jejunum. Table E, Summary of most prominent terms of GSEA analysis in jejunum.
(DOCX)
