# Supplementary material for: Early-Life Environmental Variation Affects Intestinal Microbiota and Immune Development in New-Born Piglets
Source: PLoS One. 2014 Jun 18;9(6):e100040. doi: 10.1371/journal.pone.0100040 (PMC4062469; doi:10.1371/journal.pone.0100040)
Supplement: File S1 — Supporting Tables. Table A, Pools for microbiota and transcriptomic analyses. Table B, Number of up- or down-regulated probes and genes at day 8 after birth when comparing the different treatments (T1, T2, and T3). Numbers of annotated genes are given in parentheses. Table C, Phylum level bacteria changed in T2 and T3. Table D, Summary of most prominent terms of GSEA analysis in jejunum. Table E, Summary of most prominent terms of GSEA analysis in jejunum. (DOCX) [file pone.0100040.s003.docx]

**Supporting** **information**

**Figure legends**

**Figure A**

Depicted is the experimental design, for each sow (n=16) piglets are divided over the three treatments (T1, T2, and T3). At the day of sampling 16 piglets, 1 of each sow are taken for analysis, for each treatment.

**Figure B**

In the x-axis the qPCR signal is depicted and in the y-axis the normalized expression value is depicted. Three genes are depicted IL1B (upper panel), IL6 (middle panel), and TIMP1 (lower panel).

**Supplementary Table A Pools for microbiota and transcriptomic analyses**

| T3* | T2 | T1 |
| --- | --- | --- |
| **Pool 1** | **Pool 5** | **Pool 9** |
| R2679 | R2679 | R2679 |
| R2705 | R2705 | R2705 |
| R2592 | R2592 | R2592 |
| R2704 | R2704 | R2704 |
|  |  |  |
| **Pool 2** | **Pool 6** | **Pool 10** |
| R2687 | R2687 | R2687 |
| R2423 | R2423 | R2423 |
| R2369 | R2369 | R2369 |
| R2419 | R2419 | R2419 |
|  |  |  |
| **Pool 3** | **Pool 7** | **Pool 11** |
| R2336 | R2336 | R2336 |
| R2504 | R2504 | R2504 |
| R2692 | R2692 | R2692 |
| R2514 | R2514 | R2514 |
|  |  |  |
| **Pool 4** | **Pool 8** | **Pool 12** |
| R2591 | R2591 | R2591 |
| R2701 | R2701 | R2701 |
| R2596 | R2596 | R2596 |
| R2669 | R2669 | R2669 |

***** For each treatment 4 pools are created based on the same 4 piglets from 4 sows

**Supplementary Table B Number of up- or down-regulated probes and genes at day 8 after birth when comparing the different treatments (T1, T2, and T3). Numbers of annotated genes are given in parentheses.**

| **Tissue** | **T2 vs. T1** | | **T3 vs. T1** | | **T3 vs. T2** | |
| --- | --- | --- | --- | --- | --- | --- |
|  | **Down** | **Up** | **Down** | **Up** | **Down** | **Up** |
| **Jejunum** | 63 (27) | 24 (5) | 29 (15) | 38 (16) | 6 (0) | 55 (23) |
| **Ileum** | 80 (34) | 49 (16) | 80 (24) | 76 (29) | 45 (6) | 76 (32) |
| **Blood** | 0 (0) | 0 (0) | 0 (0) | 0 (0) | 0 (0) | 0 (0) |

Abbreviations used: T1; Treatment 1,T2; Treatment 2, T3; Treatment 3.

**Supplemental Table C Phylum level bacteria changed in T2 and T3**

|  | T2 vs. T1 | T3 vs. T1 | T3 vs. T2 | ARC^1^ | | |
| --- | --- | --- | --- | --- | --- | --- |
|  | *p* value | *p* value | *p* value | T1 | T2 | T3 |
| Firmicutes | 0.34 | 0.89 | 0.11 | 80.15±3.04 | 76.86±2.01 | 79.22±0.88 |
| Proteobacteria | 0.34 | 1 | 0.11 | 11.40±2.28 | 13.45±1.41 | 12.13±0.52 |
| Bacteroidetes | 0.49 | 0.69 | 0.69 | 2.55±0.40 | 2.74±0.60 | 2.74±0.23 |
| Spirochaetes | 0.49 | 1 | 0.11 | 2.50±0.59 | 2.88±0.29 | 2.52±0.20 |
| Actinobacteria | 0.34 | 0.89 | 0.2 | 2.29±0.48 | 2.72±0.32 | 2.42±0.10 |
| Fibrobacteres | 0.34 | 0.34 | **0.03** | 0.58±0.23 | 0.70±0.11 | 0.43±0.02 |
| Deferribacteres | 0.11 | 1 | 0.34 | 0.23±0.04 | 0.29±0.06 | 0.22±0.01 |
| Verrucomicrobia | 0.11 | 1 | 0.11 | 0.19±0.03 | 0.25±0.05 | 0.18±0.01 |
| Fusobacteria | 1 | 0.34 | 0.49 | 0.10±0.01 | 0.10±0.01 | 0.11±0.01 |
| Chlamydiae | 0.49 | **0.03** | **0.03** | <0.01 | <0.01 | <0.01 |
| Planctomycetes | 0.49 | **0.03** | **0.03** | <0.01 | <0.01 | <0.01 |

^1^ ARC: average relative contribution [%] of a microbial group. Values represented means ± SD

Abbreviations used: T1; Treatment 1,T2; Treatment 2, T3; Treatment 3.

**Supplementary Table D Summary of most prominent terms of GSEA analysis in jejunum**

| **T3vsT1** | | | **T2vsT1** | | | | **T3vsT2** | | |
| --- | --- | --- | --- | --- | --- | --- | --- | --- | --- |
| **T3** | | **T1** | | **T2** | **T1** | | **T3** | | **T2** |
| DEGRADATION  (VALINE, LEUCINE, ISOLEUCINE) | | **Ribosome/translation** | | **x** | **response immune/defense/wounding** | | **x** | | **x** |
|  | | LOCOMOTORY_BEHAVIOR | |  | LOCOMOTORY_BEHAVIOR | |  | |  |
|  | | beta cell development | |  | immune cell activation | |  | |  |
|  | |  | |  | Ribosome/translation | |  | |  |
|  |  | | |  | | cell cycle (arrest/apoptosis) |  |  | |

Abbreviations used: T1; Treatment 1,T2; Treatment 2, T3; Treatment 3.

**Supplementary TableE Summary of most prominent terms of GSEA analysis in jejunum**

| **T3vsT1** | | | **T2vsT1** | | | | **T3vsT2** | | |
| --- | --- | --- | --- | --- | --- | --- | --- | --- | --- |
| **T3** | | **T1** | | **T2** | **T1** | | **T3** | | **T2** |
| membrane / receptor | | Ribosome / translation | | endosome/lysosome/membrane | chemokine/cytokine | | **x** | | **x** |
| metabole | | Immune / chemokine | | metabole (lipids / insulin ) | intestinal immunity/inflammation | |  | |  |
| apoptosis | | behavior (locomotory) | | BIOCARTA_INTEGRIN_PATHWAY | behavior (locomotory) | |  | |  |
|  | |  | | golgi | wounding | |  | |  |
|  |  | | | cell junction | | Ribosome / translation |  |  | |

Abbreviations used: T1; Treatment 1,T2; Treatment 2, T3; Treatment 3.
